# Elevated D-dimer is associated with increased 28-day mortality in acute-on-chronic liver failure in China: a retrospective study

**DOI:** 10.1186/s12876-019-0941-0

**Published:** 2019-01-31

**Authors:** Tingting Qi, Congyan Zhu, Guanting Lu, Jun Hao, Qinjun He, Yongpeng Chen, Fuyuan Zhou, Jinjun Chen, Jinlin Hou

**Affiliations:** Hepatology Unit, Department of Infectious Diseases, Nanfang Hospital, Southern Medical University, Guangzhou, China

**Keywords:** Acute-on-chronic liver failure, D-dimer, Coagulation, Fibrinolysis

## Abstract

**Background:**

Acute-on-chronic liver failure (ACLF) is a syndrome characterized by profound disrupted coagulation and fibrinolysis. Fibrinolytic marker D-dimer is increased in critically ill patients with cirrhosis which is associated with poorer prognosis. We aim to determine the potential association of D-dimer with the 28-day mortality in ACLF patients.

**Methods:**

In a single center retrospective study performed in China, we collected data of 115 patients with ACLF from October 1, 2012 to December 31, 2016. We investigated correlations between D-dimer and other laboratory tests and prognostic scores. The relationship between D-dimer and 28-day mortality was explored by smoothing plot with an adjustment for potential confounders. Logistic regression analyses with crude and adjusted models were performed to explore the association of D-dimer with 28-day mortality in ACLF patients.

**Results:**

In ACLF patients, D-dimer at admission was correlated with all prognostic scores (MELD-Na: r = 0.385, *P* < 0.001; CLIF-C ADs: r = 0.443, *P* < 0.001; CLIF-C ACLFs: r = 0.375, *P* < 0.001). A nonlinear relation between D-dimer and 28-day mortality was found with a turning point at 6.5 mg/L FEU. D-dimer level was independently associated with 28-day mortality with an adjusted odds ratio of [1.4 (1.0–1.9), *P* = 0.030] as continuous variable and [10.3 (1.3, 81.5), *P* = 0.028] as a classified variable with the cut-off of 6.5 mg/L FEU. An elevated D-dimer within the following 10 days also tended to be associated with higher risk of 28-day mortality [OR: 27.5 (0.9, 814.9), *P* = 0.055].

**Conclusions:**

Elevated D-dimer levels was associated with increased risk of 28-day mortality in patients with ACLF in China.

**Electronic supplementary material:**

The online version of this article (10.1186/s12876-019-0941-0) contains supplementary material, which is available to authorized users.

## Background

Acute-on-chronic liver failure (ACLF) is a syndrome characterized by acute decompensation of chronic liver disease associated with organ failures and high short-term mortality [1–3]. Coagulation failure reflected by elevated international normalized ratio of the prothrombin time (INR) levels was one of the most common signs of disease in ACLF patients [1, 4]. However, INR is based on the levels of procoagulant factors and not anticoagulant factors. It is regarded as an accurate marker of liver function, rather than a predictor of bleeding or thrombosis [5], which do not adequately reflect the hemostatic status in patients with liver disease.

Recent studies have highlighted the disturbances of coagulation and hemostasis (involving coagulation, platelets, and fibrinolysis) in patients with acute and chronic liver diseases [6]. Hyperfibrinolysis resulting from clotting activation has been reported in patients with cirrhosis and fulminant liver failure since 1990s [7]. Prognostic values of various biomarkers in clotting and fibrinolytic system has also been proposed [8, 9].

D-dimer is a soluble fibrin degradation product that released from crosslinked fibrin by the action of plasmin [10], which is increased in critically ill patients with cirrhosis [7, 11–13]. However, the association between D-dimer and short-term prognosis in ACLF has not been elucidated yet. The aim of this current study was to evaluate the independent effect of D-dimer on short-term prognosis of ACLF patients.

## Methods

### Study design

**Fig. 1:**
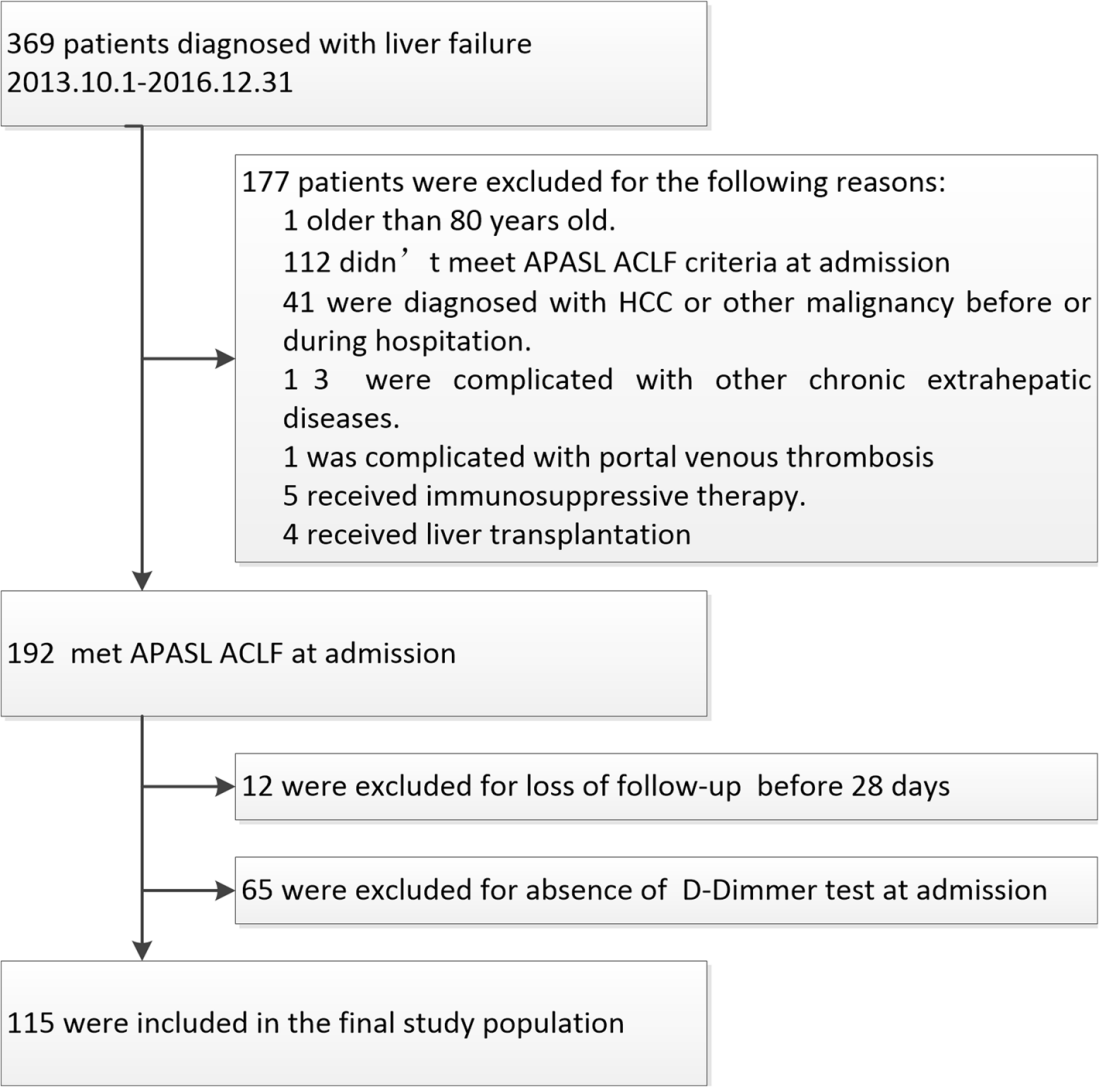
Flow-chart of patients eligible for the current study

In this single-center retrospective study, consecutive hospitalized patients admitted to the hepatology units of Nanfang hospital from October 1, 2012 to December 31, 2016 with diagnoses of liver failure at discharge or death were screened (Fig. 1). The exclusion criteria included: (1) younger than 12 or older than 80; (2) not meeting ACLF diagnostic criteria of the Asia-Pacific Association for the Study of Liver (APASL) ACLF Research Consortium [2, 3]; (3) hepatocellular carcinoma or other types of malignancies; (4) complicated with other severe chronic extrahepatic diseases; (5) complicated with portal venous thrombosis (PVT) or deep vein thrombus (DVT); (6) under immunosuppressive therapy; (7) history of liver or kidney transplantation; (8) uncertain 28-day outcome; (9) absence of D-dimer test at admission. The study protocol was approved by the Ethics Committee of Nanfang Hospital, Southern Medical University, Guangzhou, China (NFEC-201706-K4).

### Clinical data collection

Demographic data, prior history, clinical, physical examinations, laboratory tests and radiological examinations were obtained from electronic medical records. D-dimer was measured by the Sysmex CA7000 analyzers using the INNOVANCE D-dimer assay (Siemens Healthcare Diagnostics Products GmbH, Marburg, Germany). Liver cirrhosis was diagnosed by radiological evidence of liver nodularity or endoscopic signs of portal hypertension at or before admission. Survival rates were obtained through patient medical records or by direct contact with patients or their kins. The model for end-stage liver disease (MELD) [14], MELD sodium (MELD-Na) [15] scores, CLIF Consortium ACLF score (CLIF-C ACLFs) [16] and CLIF Consortium Acute Decompensation score (CLIF-C ADs) [17] were calculated. Organ failures were determined according to the CLIF Consortium Organ Failure score (CLIF-C OFs) [16]. Diagnosis of European Association for the Study of liver (EASL)-ACLF were according to CANONIC Study [1].

### Statistics analysis

Continuous variables were expressed as mean ± standard deviation (SD) if normally distributed, or median values with interquartile ranges (IQR) if not, and compared by the Student’s t-test, Mann-Whitney U test or one-way ANOVA with Kruskal-Wallis test. Nominal variables were expressed as number (percentage) and compared using Chi-square test and Fisher’s exact test when appropriate. The relationship between D-dimer and 28-day mortality was explored by smoothing plot with an adjustment for potential confounders. A two-piecewise linear regression model was applied to examine the threshold effect of the D-dimer on 28-day mortality according to the smoothing plot. We calculated odds ratios (ORs) of D-dimer for risk of 28-day mortality by logistic regression analysis. Both non-adjusted and multivariate-adjusted models [Adjust I model adjust for: age; international normalized ratio; leukocyte counts; serum bilirubin and serum creatinine. Adjust II model adjust for: age, red blood cells, hematocrit, albumin, neutrophil percentage, serum total bilirubin, international normalized ratio, C-reactive protein, hepatic encephalopathy, and ascites] were applied. Odd ratios were provided with 95% confidence intervals. The discrimination of D-dimer was determined by the area under curve (AUC) of receiver operating characteristic (ROC) curve and validated using the bootstrap resampling method with 2000 replications. The Hanley and McNeil method was used to test the significance of the differences between the AUCs of D-dimer and conventional prognostic scores. All analyses were performed with the statistical software packages R (http://www.R-project.org, The R Foundation, Vienna, Austria) and EmpowerStats (http://www.empowerstats.com, X&Y Solutions, Boston, MA, USA). A two-sided significance level of < 0.05 was used to evaluate statistical significance.

## Results

### Baseline characteristics

A total of 369 consecutive patients were screened, of whom 192 met APASL ACLF at admission. Seventy seven cases were further excluded, 12 for loss of follow-up before 28 days and 65 were due to lack of D-dimer test results at admission. Finally, 115 patients were enrolled in the current study (Fig. 1).

Baseline characteristics and clinical outcomes were shown in Table 1. Overall, 94.5% of the subjects had Hepatitis B virus (HBV) related chronic liver disease, 54.8% were diagnosed with cirrhosis, 94.8% were combined with clinical ascites, and 27.0% suffered hepatic encephalopathy. D-dimer at admission was 3.8 ± 2.9 mg/L FEU. Of note, 51.3% of the subjects also met EASL-CLIF ACLF criteria, Liver failure was the most frequent organ failure (86.1%), followed by coagulation failure (37.4%) according to CLIF-OF criteria. The 28-day and 90-day mortality rate were 35.7 and 47.0%, respectively (Table 1).

**Table 1:** Baseline variables and clinical outcomes in subjects enrolled

Variable	Overall (*n* = 115)
Age (y)	44.6 (11.3)
Gender (male)	102 (88.7%)
Etiology	
HBV-related (%)	109 (94.8%)
Cirrhosis (%)	63 (54.8%)
Ascites (%)	109 (94.8%)
Hepatic encephalopathy (%)	31 (27.0%)
Laboratory tests	
Red blood cell count	3.9 (0.8)
Hemoglobin (g/L)	118.7 (23.1)
Hematocrit (%)	33.6 (6.7)
Platelet count (×10^9^/L)	109.6 (52.9)
Leucocyte count (×10^9^/L)	8.9 (4.3)
Neutrophil percentage (%)	69.8 (11.0)
Lymphocyte percentage (%)	19.1 (8.9)
Alanine aminotransferase (U/L)	188.0 (60.2–622.8)
Aspartate aminotransferase (U/L)	172.4 (97.0–395.5)
Serum bilirubin (μmol/L)	392.1 (275.8–529.2)
Albumin (g/L)	30.8 (5.1)
Serum creatinine (μmol/L)	77.0 (60.5–100.5)
Urea nitrogen (μmol/L)	3.8 (2.9–6.1)
Serum sodium (mmol/L)	136.7 (4.7)
C-reactive protein (mg/L)	11.6 (6.9–17.3)
International normalized ratio	2.5 (0.8)
D-dimer (mg/L FEU)	3.8 (2.9)
Fibrinogen (g/L)	1.3 (0.4)
HBV DNA (> 20,000 IU/ml, %)	65 (74.7%)
Organ failure (%)	
Liver	99 (86.1%)
Coagulation	43 (37.4%)
Brain	12 (10.4%)
Kidney	11 (9.6%)
Circulation	3 (2.6%)
Respiration	3 (2.6%)
EASL-ACLF criteria (%)	59 (51.3%)
EASL-ACLF Grade (%)	
Grade-0	56 (48.7%)
Grade-1	7 (6.1%)
Grade-2	42 (36.5%)
Grade-3	10 (8.7%)
Prognostic scores	
MELD score	27.2 (6.3)
MELD-Na score	29.1 (9.0)
CLIF-C ACLFs	43.0 (8.3)
CLIF-C ADs	58.0 (9.9)
Mortality (%)	
28-day	41 (35.7%)
90-day	54 (47.0%)

Particularly, we also compared baseline characteristics and short-term prognosis between 115 patient enrolled and 65 cases who were excluded due to lack of D-dimer measurement at admission. As showed in Additional file 1 (Table S1), all baseline variables were comparable between these two groups of subjects, except for higher percentages of hepatic encephalopathy (27.0% vs. 10.8%, *P* = 0.011), brain failure (10.4% vs. 1.5%, *P* = 0.034), and proportion of patients who met EASL ACLF criteria as well (51.3% vs. 35.4%, *P* = 0.039) among those with D-dimer test at admission. All prognostic scores, 28-day mortality and 90-day mortality were similar between these two groups.

### D-dimer was correlated with multi-organ injuries

The correlations between baseline D-dimer level and other laboratory data were explored (Table 2). D-dimer was inversely correlated with log transformed alanine aminotransferase levels (r = − 0.284, *P* = 0.002), but not correlated with total bilirubin (TB) levels (r = − 0.124, *P* = 0.189). D-dimer level was positively correlated with INR level (r = 0.234, *P* = 0.012) and inversely correlated with platelet counts (r = − 0.353, *P* < 0.001). We also recorded significantly positive correlations between D-dimer and serum creatinine (r = 0.277, *P* = 0.003), and urea nitrogen as well (r = 0.385, *P* < 0.001). Positive correlations were also detected between D-dimer levels and leukocyte counts (r = 0.292, *P* = 0.002), percentages of neutrophils (r = 0.250, *P* = 0.007) and C-reactive protein levels (r = 0.38, *P* < 0.001).

**Table 2:** Associations of clinical parameters and prognostic scoring systems with D-dimer

Variable	Correlation coefficient	*P* value
	with D-dimer (r)	
Age (y)	0.168	0.073
Laboratory data		
Hemoglobin	−0.298	0.001
Hematocrit	−0.271	0.004
Platelet count	−0.353	< 0.001
Leukocyte count	0.292	0.002
Neutrophil percentage	0.25	0.007
C-reactive protein	0.38	< 0.001
Log transformed alanine aminotransferase	−0.284	0.002
Log transformed aspartate aminotransferase	−0.16	0.088
Log transformed total bilirubin	−0.124	0.189
Serum creatinine	0.277	0.003
Urea nitrogen	0.385	< 0.001
International normalized ratio	0.234	0.012
Fibrinogen	−0.29	0.008
Prognostic scores		
MELD	0.297	0.001
MELD-Na	0.385	< 0.001
CLIF-C ADs	0.443	< 0.001
CLIF-C ACLFs	0.375	< 0.001

**Fig. 2:**
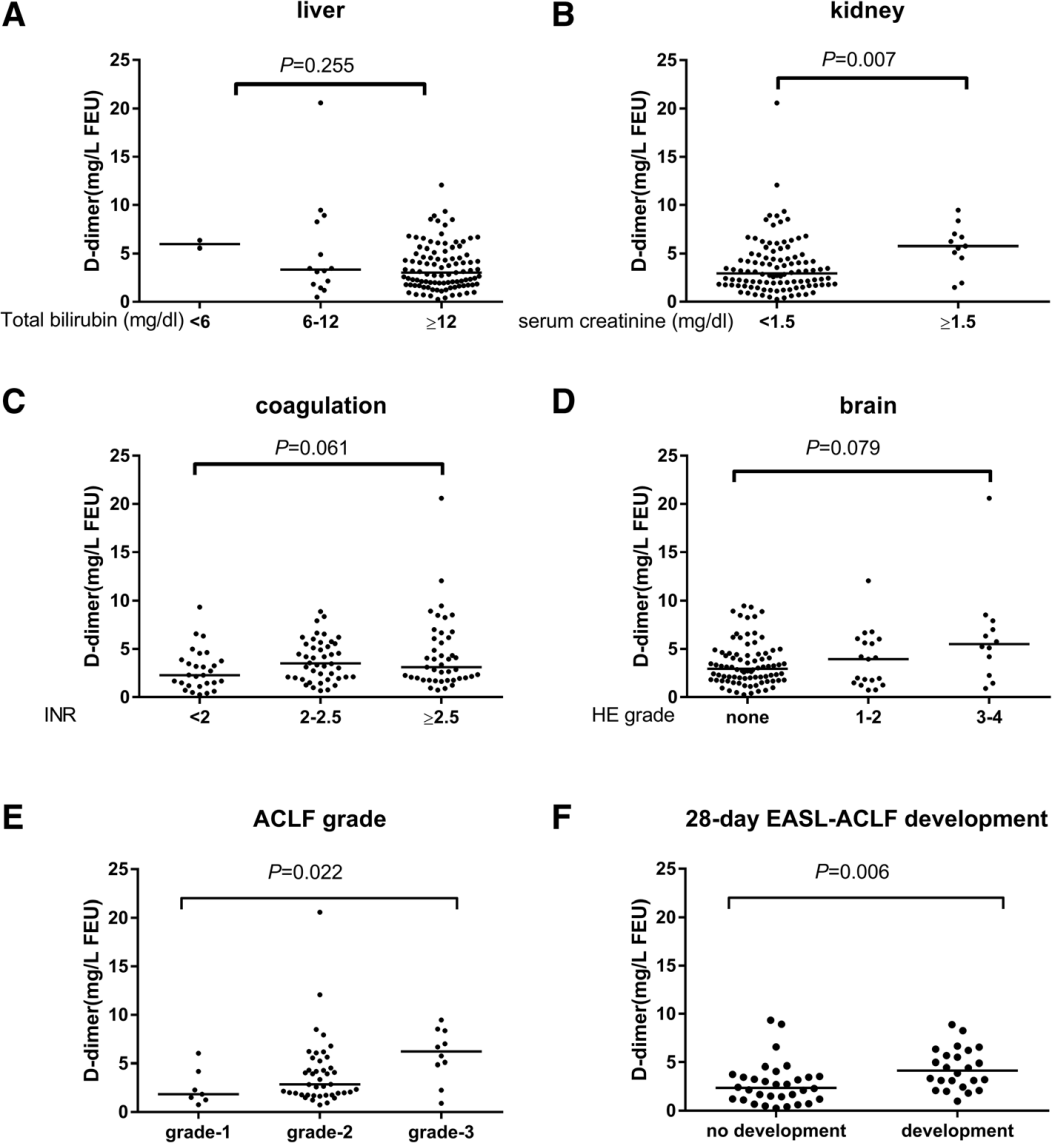
D-dimer levels and organs injury. Comparisons of D-dimer levels among subjects within subgroups of ACLF subjects according to CLIF-OF score: **a** liver; **b** kidney; **c** coagulation; **d** brain and **e** ACLF grade. **f** D-dimer levels between subjects with and without EASL ACLF development in subjects with no ACLF at admission

D-dimer levels at admission were further compared among subjects with various degrees of organs injury according to CLIF-OF score system (Fig. 2). D-dimer levels were unanticipated comparable in subjects with different levels of TB [TB < 6 mg/dl, medium (interquartile range), 5.9 (5.6, 6.3), 6-12 mg/dl: 3.3 (1.7, 8.4), ≥12 mg/dl: 3.0 (1.8, 5.0), *P* = 0.255] (Fig. 2a). D-dimer was higher in subjects with kidney failure [creatinine < 1.5 mg/dl: 3.0 (1.8, 4.7), ≥1.5 mg/dl: 5.8 (4.6, 7.0), *P* = 0.007] (Fig. 2b). D-dimer also marginally increased in subjects with severer coagulation and brain injuries [INR < 2: 2.3 (1.3, 3.9), 2–2.5: 3.5 (2.0, 5.5), ≥2.5: 3.1 (2.0, 6.0), *P* = 0.061; no hepatic encephalopathy: 3.0 (1.8, 4.5), 1–2 grade encephalopathy: 3.9 (1.5, 6.0); 3–4 grade encephalopathy: 5.5 (2.7, 7.7), *P* = 0.079] (Fig. 2c-d). In addition, D-dimer level varies significantly among EASL-ACLF subjects with different ACLF grades [grade 1: 1.8 (1.3, 4.2), grade 2: 2.9 (1.8, 5.3), grade 3:6.2 (4.2, 8.4), *P* = 0.022] (Fig. 2e). Among subjects who didn’t met EASL-ACLF criteria at admission, D-dimer was higher in those who developed EASL-ACLF within 28 days than those without EASL ACLF development [4.2 (2.6, 6.1) vs. 2.4 (1.2, 3.5), *P* = 0.006] (Fig. 2f).

**Fig. 3:**
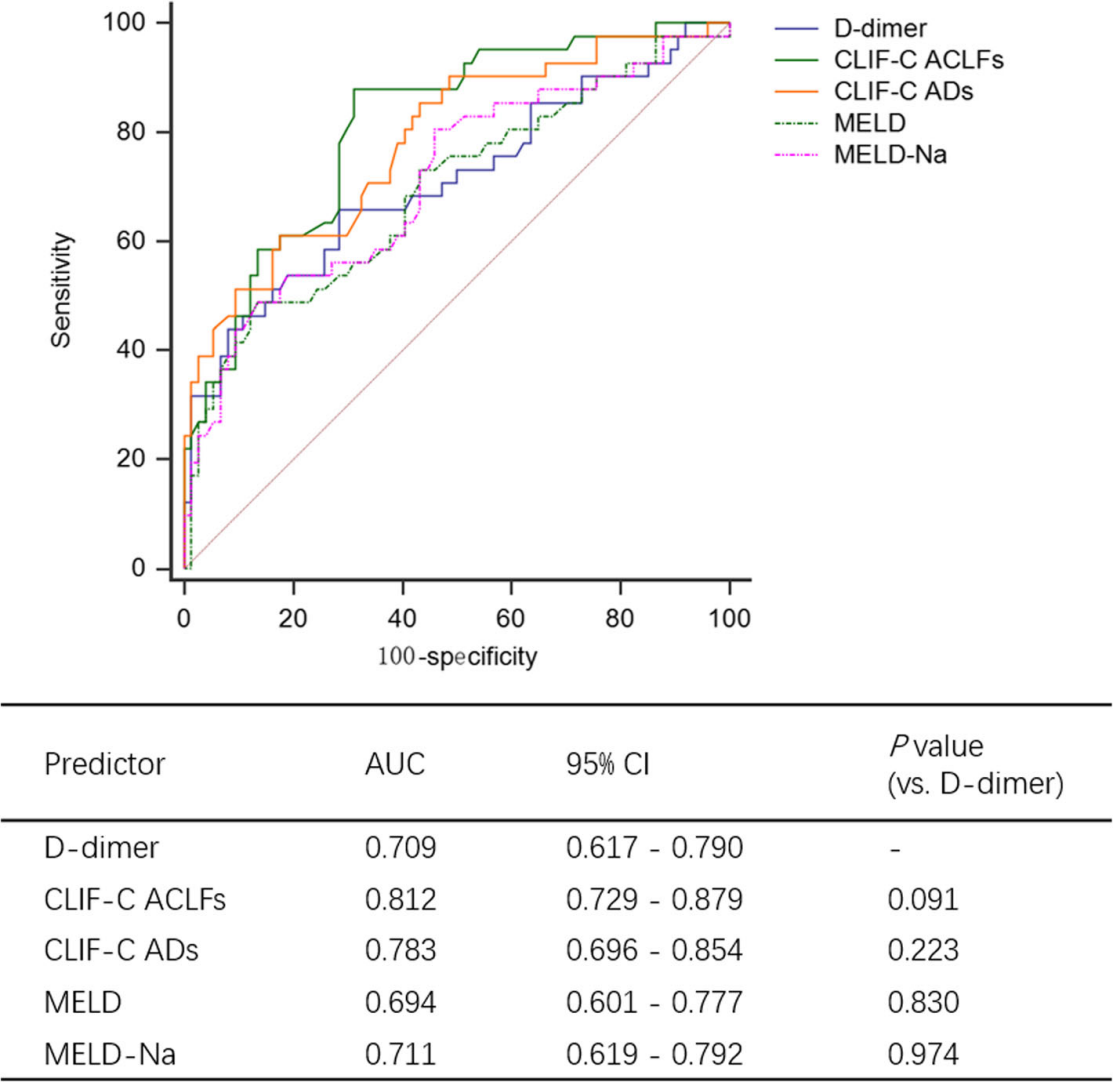
Comparison of AUROC of D-dimer and conventional prognostic scores in predicting 28-day mortality of ACLF patients

Furthermore, D-dimer levels positively correlated with all prognostic predictive scores (MELD: r = 0.297, *P* = 0.001; MELD-Na: r = 0.385, *P* < 0.001; CLIF-OF: r = 0.31, *P* < 0.001; CLIF-C ADs: r = 0.443, *P* < 0.001; CLIF-C ACLFs: r = 0.375, *P* < 0.001, Table 2). The predictive value of D-dimer and other prognostic scores were examined by ROC curve analysis and comparisons. The AUC for D-dimer alone was 0.709 (0.617–0.790) [0.711 (0.605–0.825) in bootstrap validation], which was comparable with CLIF-C ACLF score [0.812 (0.729–0.879), *P* = 0.091], CLIF-C AD score [0.783 (0.696–0.854), *P* = 0.223], MELD score [0.694 (0.601–0.777), *P* = 0.830] and MELD-Na score [0.711 (0.619–0.792), *P* = 0.974] (Fig. 3).

### D-dimer independently correlated with 28-day mortality in ACLF

**Fig. 4:**
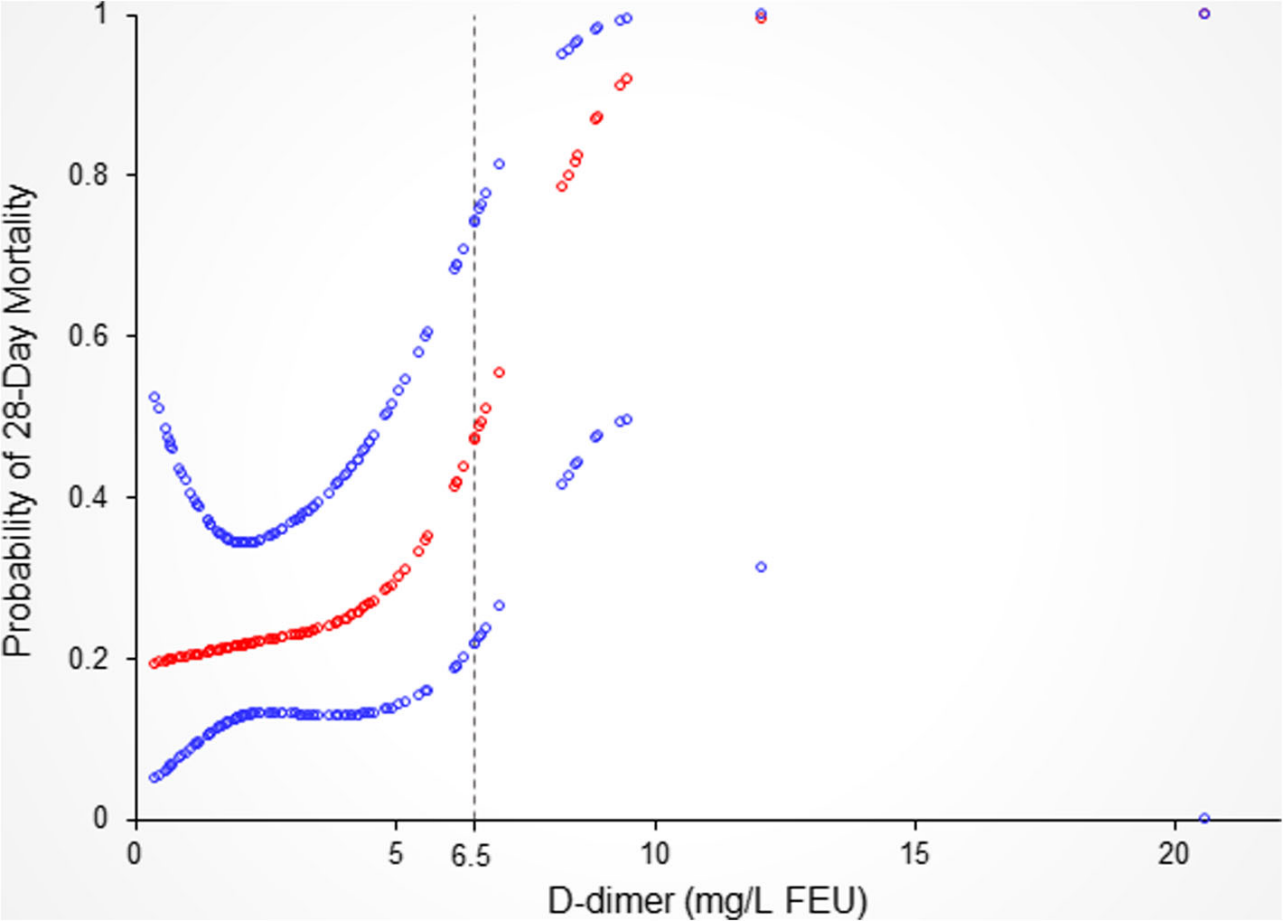
Relationship between D-dimer at admission and 28-day mortality*. The nonlinear relationship between D-dimer and probability of death within 28 days was observed, and a threshold D-dimer of 6.5 mg/L FEU existed for increased probability of 28-day mortality. *Adjusted for age, red blood cells, hematocrit, albumin, neutrophil percentage, TB, INR, C-reactive protein, hepatic encephalopathy, and ascites

Next, we explored the correlation between D-dimer levels and 28-day prognosis. A nonlinear relationship between D-dimer levels and 28-day mortality with adjustment of possible confounders was detected (Fig. 4). With a D-dimer level < 6.5 mg/L FEU, the estimated dose-response curve was consistent with a horizontal line (β = 1.0, 95% CI = 0.6 to 1.6, *P* = 0.976). However, the 28-day mortality increased with increasing D-dimer when exceeded the turning point (D-dimer> 6.5 mg/L FEU), with the correlation coefficient (β) of 5.7 (1.3, 24.3) (*P* = 0.020) (Fig. 4, Table 3). Baseline characteristics between subjects with different D-dimer levels were shown in Additional file 1 (Table S2).

**Table 3:** Threshold effect analysis of D-dimer level on 28-day outcome

	Crude ß/OR (95%CI) *P* value	^a^Adjusted ß/OR (95%CI) *P* value
D-dimer< 6.5 mg/L FEU	1.3 (1.0, 1.6) 0.040	1.0 (0.6, 1.6) 0.976
D-dimer≥6.5 mg/L FEU	2.7 (0.9, 7.8) 0.070	5.7 (1.3, 24.3) 0.020
Logarithmic likelihood ratio test *P* value	0.168	0.035

^a^Adjusted: age, red blood cells, hematocrit, albumin, neutrophil percentage, serum total bilirubin, international normalized ratio, C-reactive protein, hepatic encephalopathy, and ascites

The association between D-dimer and 28-day outcome was further explored by logistic regression analysis (Table 4). As a continuous variable, baseline D-dimer level was associated with increased 28-day morality in both non-adjusted model [β: 1.4 (1.2, 1.7), *P* < 0.001] and adjusted models [β: 1.4, (1.0, 1.9), *P* = 0.03]. When included as a binary variable, a D-dimer level ≥ 6.5 mg/L FEU was associated with significant increased risk of 28-day mortality compared to those with D-dimer level < 6.5 mg/L FEU [adjusted odds ratio: 10.3 (1.3–81.5), Table 4].

**Table 4:** Association between D-dimer and 28-day outcome

	Non-adjusted	Adjust I	Adjust II
D-dimer	1.4 (1.2, 1.7) < 0.001	1.4 (1.1, 1.7) 0.010	1.4 (1.0, 1.9) 0.030
D-dimer			
< 6.5 mg/L FEU	1	1	1
≥6.5 mg/L FEU	8.1 (2.4, 27.1) 0.001	11.9 (2.3, 63.3) 0.004	10.3 (1.3, 81.5) 0.028
delta D-dimer^a^			
≥0 (stable or decreased)	1	1	1
< 0 (elevated)	6.3 (1.2, 32.3) 0.026	9.9 (1.5, 65.0) 0.017	27.5 (0.9, 814.9) 0.055

Non-adjusted model adjust for: None

Adjust I model adjust for: age; international normalized ratio; leukocyte counts; serum bilirubin and serum creatinine

Adjust II model adjust for: age, red blood cells, hematocrit, albumin, neutrophil percentage, serum total bilirubin, international normalized ratio, C-reactive protein, hepatic encephalopathy, and ascites

^a^delta D-dimer was analyzed in 51 subjects with repeated D-dimer test at day 4–10 after admission. delta D-dimer = D-dimer (at admission)-D-dimer (at day 4–10)

We additionally evaluated the correlation between the dynamics of D-dimer level and 28-day mortality. Among 51 subjects with repeated D-dimer test at day 4 to 10 after admission, those with increased D-dimer levels tend to have higher risk of 28-day morality [adjusted odds ratio: 27.5 (0.9–814.9)] compared with subjects with stable or decreased D-dimer levels (Table 4).

The association between D-dimer levels and 28-day morality across subgroups according to underlying cirrhosis, ACLF diagnosed according to EASL criteria, coagulation failure (defined as INR ≥ 2.5), hepatic encephalopathy and leukocyte count levels (< 4 × 10^9^/L or > 12 × 10^9^/L) were studied. As shown in Table 5, we detected no significant subgroup interactions.

**Table 5:** Odds ratios for 28-day mortality per unit increase in D-dimer in subgroups of patients

	No. of events	Non-adjusted	*P* for interaction	Adjust I	*P* for interaction	Adjust II	*P* for interaction
Cirrhosis							
yes	63	1.4 (1.1, 1.8) 0.004	0.948	1.4 (1.1, 1.9) 0.016	0.479	1.4 (1.0, 2.0) 0.088	0.866
no	52	1.4 (1.1, 1.9) 0.018		1.2 (0.8, 1.8) 0.361		1.5 (0.9, 2.4) 0.138	
EASL-ACLF							
yes	59	1.6 (1.2, 2.1) 0.002	0.452	1.4 (1.0, 2.0) 0.039	0.797	1.4 (0.9, 2.3) 0.128	0.886
no	56	1.4 (1.0, 1.8) 0.040		1.3 (0.9, 1.9) 0.105		1.4 (0.9, 2.1) 0.168	
Coagulation failure							
yes	43	1.4 (1.0, 1.9) 0.024	0.688	1.2 (0.8, 1.6) 0.328	0.259	1.3 (0.7, 2.4) 0.322	0.623
no	72	1.5 (1.2, 2.0) 0.003		1.5 (1.1, 2.1) 0.009		1.6 (1.0, 2.8) 0.060	
Hepatic encephalopathy							
yes	31	1.3 (1.0, 1.8) 0.087	0.496	1.1 (0.8, 1.6) 0.648	0.161	1.1 (0.7, 1.6) 0.797	0.202
no	84	1.5 (1.2, 1.9) 0.001		1.5 (1.1, 2.0) 0.005		1.6 (1.1, 2.3) 0.015	
leukocyte counts							
< 4 × 10^9^/L or > 12 × 10^9^/L	28	1.7 (1.1, 2.6) 0.010	0.263	1.7 (1.0, 2.9) 0.054	0.254	2.1 (0.9, 4.5) 0.075	0.156
4 × 10^9^/L - 12 × 10^9^/L	87	1.3 (1.1, 1.6) 0.007		1.2 (0.9, 1.6) 0.149		1.2 (0.9, 1.8) 0.256	

Non-adjusted model adjust for: None

Adjust I model adjust for: age; international normalized ratio; leukocyte counts; serum bilirubin and serum creatinine

Adjust II model adjust for: age, red blood cells, hematocrit, albumin, neutrophil percentage, serum total bilirubin, international normalized ratio, C-reactive protein, hepatic encephalopathy, and ascites

## Discussion

In the present study we investigated the fibrinolytic biomarker D-dimer in patients with ACLF. The most important finding was that D-dimer levels correlated with 28-day mortality in ACLF patients in both non-adjusted and adjusted models. Interestingly, when adjusting for the possible confounders related to D-dimer levels and short-term mortality, a non-linear relationship was observed between D-dimer and 28-day mortality. Threshold effect was then found at the turning point when D-dimer level reached 6.5 mg/L FEU, indicating that the risk for 28-day mortality increased in ACLF patients when they had a D-dimer level higher than 6.5 mg/L FEU. These results emphasize the importance of coagulation and fibrinolysis in the progression of ACLF.

The origin of elevated D-dimer in ACLF is not clear. Venous thromboembolism including portal vein thrombosis are increasingly identified in patients with cirrhosis [18, 19]. However, there is no evidence that the development of PVT is responsible for further progression of liver disease [18]. Moreover, with 94.8% of previous compensated chronic HBV related liver disease patients with ACLF, the incidence of PVT or DVT in the current cohort was very low. We only excluded one patients for PVT at enrollment. Therefore, the elevated D-dimer in ACLF patients are less likely derived from venous thromboembolism.

Infection and sepsis is highly prevalent in ACLF patients and correlated with poorer prognosis in ACLF [20, 21]. Activation of coagulation in concert with inflammatory activation can result in microvascular thrombosis, which contributes to multiple organ failure in patients with severe sepsis [22]. Indeed, when stratified according to leukocytes counts, the odds ratios of D-dimer in patients with leukocyte cells count < 4 × 10^9^/L or > 12 × 10^9^/L seem to be higher [2.1 (0.9–4.5)] than those with normal leukocyte count [1.2 (0.9, 1.8)], although the interaction effect was not significant (*P* = 0.1556) which might attribute to limited sample size. Moreover, the correlations between D-dimer and multi-organ injuries supported systematic inflammation. In this circumstances, D-dimer is not only a marker of activation of fibrinolysis, but may also indicating profound systematic inflammation.

Hepatic hyper-coagulation and fibrin deposition were also elucidated in explanted liver tissue and mice models of acute liver failure [23–25]. Progressive occlusion of portal microvasculature by microthrombi has been proposed in advanced cirrhosis [26, 27], non-cirrhotic intrahepatic portal hypertension [28] and hepatic necrosis in liver failure [9]. Therefore intrahepatic hyper-coagulation which leads to secondary fibrinolysis might attribute to the elevated plasma D-dimer in ACLF. However, most previous clinical research aforementioned were based on cohort mainly composed of alcoholic liver disease, while the coagulation and fibrinolysis status of HBV related liver disease which constituted the majority of current cohort was seldom reported. Further studies were needed to provide evidence about the association of D-dimer, systematic inflammation, and intrahepatic hyper-coagulation.

Ascites fluid is another possible origin for hyper-fibrinolysis in advanced liver disease [29]. There were 94.8% patients in current cohort had ascites, all of which were the first episode of ascites within four weeks according to APASL ACLF definition [2, 3]. We had adjusted ascites as a confounder in full adjusted models. Therefore, despite ascites might partly explains the elevated plasma D-dimer level, the prognostic value of D-dimer is independent of the presence of ascites. Whether the fibrinolytic activity in ascites varies among patients with distinct prognosis remains to be elucidated.

ACLF is an extraordinarily dynamic syndrome [30]. Here we analyzed 51 subjects with repeated D-dimer test within the first 10 days after admission. Patients with a stable or elevated D-dimer tend to have a higher mortality rate [OR: 27.5 (0.9, 814.9)] in full adjusted model, but with wider confidence interval due to the limited sample size. This result indicating the necessity of monitoring D-dimer level in ACLF patients in clinical practice.

The major limitation of this study is that although D-dimer is a fibrinolytic marker that easy to be performed in clinical practice, D-dimer results are not comparable among various assays, even among those using similar formats [10]. Whether D-dimer tested with other methods have the similar predictive value remains to be clear. Secondly, due to the retrospective desire, we can not depict the overall perspective of coagulation and fibrinolysis in ACLF, whether other fibrin degradation products have the same effect remains to be clear. Thirdly, the majority of patients in current cohort was HBV-related, whether D-dimer independently predicts poorer short-term prognosis in ACLF related to other etiologies remained to be determined. Besides, there were 65 cases excluded due to lack of D-dimer measurement at admission which may raise selection bias. However, we demonstrated that all baseline characteristics of these patients were comparable to those enrolled in the study, except lower proportion of hepatic encephalopathy and brain failure. Since we detect no significant subgroup interactions between cases with and without encephalopathy, these lost cases had limited effect on the evaluation of independent effect of D-dimer on short-term prognosis of ACLF.

## Conclusion

This study demonstrated that fibrin degradation products D-dimer correlated with short-term prognosis in ACLF. Patients with elevated D-dimer levels at admission had an increased risk of 28-day mortality. Monitoring D-dimer level provides help in predicting short-term prognosis in ACLF patients.

## Additional file

Additional file 1: **Table S1.** Baseline variables and clinical outcomes in subjects with and without D-dimer measurement at admission. **Table S2.** Comparisons of baseline variables and clinical outcomes between patients with different D-dimer levels. (DOCX 26 kb)

## Abbreviations

ACLF: Acute-on-chronic liver failure
APASL: Asia-Pacific Association for the Study of Liver
AUC: Area under the receiver operating characteristic curve
CLIF-C ACLFs: CLIF Consortium ACLF score
CLIF-C ADs: CLIF Consortium Acute Decompensation score
CLIF-C OFs: CLIF Consortium Organ Failure score
DVT: Deep vein thrombus
EASL: European Association for the Study of Liver
HBV: Hepatitis B virus
INR: International normalized ratio of the prothrombin time
MELD: Model for end-stage liver disease
PVT: Portal venous thrombosis
ROC: Receiver operating characteristic curve
TB: Total bilirubin
