# Supplementary material for: Elevated D-dimer is associated with increased 28-day mortality in acute-on-chronic liver failure in China: a retrospective study
Source: BMC Gastroenterol. 2019 Jan 31;19:20. doi: 10.1186/s12876-019-0941-0 (PMC6357416; doi:10.1186/s12876-019-0941-0)
Supplement: Supplementary file 1 — Table S1. Baseline variables and clinical outcomes in subjects with and without D-dimer measurement at admission. Table S2. Comparisons of baseline variables and clinical outcomes between patients with different D-dimer levels. (DOCX 26 kb) [file 12876_2019_941_MOESM1_ESM.docx]

Table S1. Baseline variables and clinical outcomes in subjects with and without D-dimer measurement at admission.

| **Variable** | D-Dimer available | D-dimer unavailable | *P*-value |
| --- | --- | --- | --- |
|  | (n=115) | (n=65) |  |
| **Age (y)** | 44.6 (11.3) | 46.7 (11.3) | 0.227 |
| **Gender (male)** | 102 (88.7%) | 57 (87.7%) | 0.84 |
| **Etiology** |  |  |  |
| HBV-related (%) | 109 (94.8%) | 62 (95.4%) | 0.859 |
| **Cirrhosis (%)** | 63 (54.8%) | 36 (55.4%) | 0.938 |
| **Ascites (%)** | 109 (94.8%) | 62 (95.4%) | 0.859 |
| **Hepatic encephalopathy (%)** | 31 (27.0%) | 7 (10.8%) | 0.011 |
| **Laboratory tests** |  |  |  |
| Red blood cell count | 3.9 (0.8) | 4.0 (0.9) | 0.266 |
| Hemoglobin (g/L) | 118.7 (23.1) | 123.6 (21.2) | 0.165 |
| Hematocrit (%) | 33.6 (6.7) | 34.8 (5.9) | 0.231 |
| Platelet count (×10^9^/L) | 109.6 (52.9) | 114.7 (56.5) | 0.541 |
| Leucocyte count (×10^9^/L) | 8.9 (4.3) | 8.5 (4.5) | 0.578 |
| Neutrophil percentage (%) | 69.8 (11.0) | 69.8 (10.2) | 0.966 |
| Lymphocyte percentage (%) | 19.1 (8.9) | 19.3 (9.0) | 0.874 |
| Alanine aminotransferase (U/L) | 188.0 (60.2-622.8) | 220.0 (94.0-812.0) | 0.198 |
| Aspartate aminotransferase (U/L) | 172.4 (97.0-395.5) | 201.8 (110.0-622.7) | 0.092 |
| Serum bilirubin (μmol/L) | 392.1 (275.8-529.2) | 350.6 (237.7-497.9) | 0.222 |
| Albumin (g/L) | 30.8 (5.1) | 29.7 (5.6) | 0.165 |
| Serum creatinine (μmol/L) | 77.0 (60.5-100.5) | 79.0 (69.0-101.0) | 0.357 |
| Urea nitrogen (μmol/L) | 3.8 (2.9- 6.1) | 4.1 (2.8-5.5) | 0.848 |
| Serum sodium (mmol/L) | 136.7 (4.7) | 136.3 (4.6) | 0.55 |
| C-reactive protein (mg/L) | 11.6 (6.9- 17.3) | 12.8 (8.0-21.3) | 0.458 |
| International normalized ratio | 2.5 (0.8) | 2.5 (0.9) | 0.788 |
| Fibrinogen (g/L) | 1.3 (0.4) | 1.2 (0.3) | 0.475 |
| HBV DNA (>20,000IU/ml, %) | 65 (74.7%) | 33 (68.8%) | 0.457 |
| **Organ failure (%)** |  |  |  |
| Liver | 99 (86.1%) | 54 (83.1%) | 0.587 |
| Coagulation | 43 (37.4%) | 21 (32.3%) | 0.494 |
| Brain | 12 (10.4%) | 1 ( 1.5%) | 0.034 |
| Kidney | 11 (9.6%) | 3 ( 4.6%) | 0.234 |
| Circulation | 3 (2.6%) | 1 ( 1.5%) | 0.64 |
| Respiration | 3 (2.6%) | 1 ( 1.5%) | 0.64 |
| **EASL-ACLF criteria (%)** | 59 (51.3%) | 23 (35.4%) | 0.039 |
| **EASL-ACLF Grade (%)** |  |  |  |
| Grade-0 | 56 (48.7%) | 42 (64.6%) | 0.211 |
| Grade-1 | 7 (6.1%) | 3 ( 4.6%) |  |
| Grade-2 | 42 (36.5%) | 15 (23.1%) |  |
| Grade-3 | 10 (8.7%) | 5 ( 7.7%) |  |
| **Prognostic scores** |  |  |  |
| MELD score | 27.2 (6.3) | 26.9 (5.6) | 0.747 |
| MELD-Na score | 29.1 (9.0) | 28.9 (7.2) | 0.855 |
| CLIF-C ACLFs | 43.0 (8.3) | 41.6 (7.9) | 0.262 |
| CLIF-C ADs | 58.0 (9.9) | 58.1 (9.0) | 0.924 |
| **Mortality (%)** |  |  |  |
| 28-day | 41 (35.7%) | 24 (36.9%) | 0.865 |
| 90-day | 54 (47.0%) | 34 (52.3%) | 0.49 |

Table S2. Comparisons of baseline variables and clinical outcomes between patients with different D-dimer levels.

| **Variable** | D-dimer<6.5 mg/L FEU(n=98) | D-dimer ≥6.5mg/L FEU (n=17) | *P*-value |
| --- | --- | --- | --- |
| **Age (y)** | 44.4 (11.1) | 45.9 (12.8) | 0.617 |
| **Gender (male)** | 87 (88.8%) | 15 (88.2%) | 0.948 |
| **Etiology** |  |  |  |
| HBV-related (%) | 93 (94.9%) | 16 (94.1%) | 0.894 |
| **Cirrhosis (%)** | 52 (53.1%) | 11 (64.7%) | 0.373 |
| **Ascites (%)** | 93 (94.9%) | 16 (94.1%) | 0.894 |
| **Hepatic encephalopathy (%)** | 24 (24.5%) | 7 (41.2%) | 0.152 |
| **Laboratory tests** |  |  |  |
| Red blood cell count | 4.0 (0.8) | 3.6 (0.9) | 0.115 |
| Hemoglobin (g/L) | 119.8 (23.7) | 112.6 (18.1) | 0.235 |
| Hematocrit (%) | 33.9 (7.0) | 31.9 (5.2) | 0.261 |
| Platelet count (×10^9^/L) | 114.2 (53.9) | 82.6 (38.3) | 0.023 |
| Leucocyte count (×10^9^/L) | 8.4 (3.9) | 11.6 (5.9) | 0.005 |
| Neutrophil percentage (%) | 68.4 (10.9) | 77.6 ( 8.4) | 0.001 |
| Lymphocyte percentage (%) | 20.2 (8.9) | 12.7 (6.0) | 0.001 |
| Alanine aminotransferase (U/L) | 218.0 (70.0-661.0) | 62.0 (40.8-247.0) | 0.018 |
| Aspartate aminotransferase (U/L) | 182.2 (104.2-396.2) | 97.0 (68.0-170.0) | 0.074 |
| Serum bilirubin (μmol/L) | 382.6 (276.8-538.5) | 426.9 (208.3-528.2) | 0.653 |
| Albumin (g/L) | 31.0 (5.0) | 29.6 (5.6) | 0.31 |
| Serum creatinine (μmol/L) | 74.0 (59.0- 93.8) | 95.0 (70.0-131.0) | 0.018 |
| Urea nitrogen (μmol/L) | 3.7 (2.8- 5.3) | 6.3 (3.4-15.5) | 0.011 |
| Serum sodium (mmol/L) | 137.0 (4.2) | 135.1 (6.8) | 0.117 |
| C-reactive protein (mg/L) | 10.7 ( 6.9-14.8) | 19.8 (12.7-30.1) | 0.007 |
| International normalized ratio | 2.4 (0.8) | 2.8 (1.0) | 0.105 |
| D-dimer (mg/L FEU) |  |  |  |
| Fibrinogen (g/L) | 1.3 (0.4) | 1.1 (0.4) | 0.145 |
| HBV DNA (>20,000IU/ml, %) | 55 (73.3%) | 10 (83.3%) | 0.459 |
| **Organ failure (%)** |  |  |  |
| Liver | 86 (87.8%) | 13 (76.5%) | 0.215 |
| Coagulation | 33 (33.7%) | 10 (58.8%) | 0.048 |
| Brain | 8 (8.2%) | 4 (23.5%) | 0.056 |
| Kidney | 7 (7.1%) | 4 (23.5%) | 0.034 |
| Circulation | 1 (1.0%) | 2 (11.8%) | 0.057 |
| Respiration | 2 (2.0%) | 1 (5.9%) | 0.384 |
| **EASL-ACLF criteria (%)** | 49 (50.0%) | 10 (58.8%) | 0.502 |
| **EASL-ACLF Grade (%)** |  |  |  |
| Grade-0 | 49 (50.0%) | 7 (41.2%) | 0.009 |
| Grade-1 | 7 (7.1%) | 0 (0.0%) |  |
| Grade-2 | 37 (37.8%) | 5 (29.4%) |  |
| Grade-3 | 5 (5.1%) | 5 (29.4%) |  |
| **Prognostic scores** |  |  |  |
| MELD score | 26.6 (6.0) | 30.9 (6.9) | 0.009 |
| MELD-Na score | 28.1 ( 8.1) | 35.0 (11.9) | 0.003 |
| CLIF-C ACLFs | 42.0 ( 7.2) | 48.9 (11.7) | 0.001 |
| CLIF-C ADs | 56.8 ( 8.6) | 65.0 (13.6) | 0.001 |
| **Mortality (%)** |  |  |  |
| 28-day | 28 (28.6%) | 13 (76.5%) | <0.001 |
| 90-day | 40 (40.8%) | 14 (82.4%) | 0.002 |
